# A Survey on Real‐World Transurethral Surgery Procedures for Bladder Pain Syndrome and Interstitial Cystitis

**DOI:** 10.1111/luts.70058

**Published:** 2026-03-11

**Authors:** Niimi Aya, Akiyama Yoshiyuki, Furuta Akira, Matsuo Tomohiro, Kitta Takeya, Otsuka Atsushi, Mitsui Takahiko, Masumori Naoya, Matsukawa Yoshihisa, Torimoto Kazumasa, Kinjo Manami, Chiba Hiroki, Nomiya Akira, Maeda Daichi, Homma Yukio

**Affiliations:** ^1^ Department of Urology, Graduate School of Medicine The University of Tokyo Tokyo Japan; ^2^ Department of Urology Shinshu University School of Medicine Matsumoto Japan; ^3^ Department of Urology Jikei University School of Medicine Tokyo Japan; ^4^ Department of Urology Nagasaki University Graduate School of Biomedical Sciences Nagasaki Japan; ^5^ Department of Renal and Urologic Surgery Asahikawa Medical University Asahikawa Japan; ^6^ Department of Urology Iwata City Hospital Shizuoka Japan; ^7^ Department of Urology, Interdisciplinary Graduate School of Medicine and Engineering University of Yamanashi Yamanashi Japan; ^8^ Department of Urology Sapporo Medical University School of Medicine Sapporo Japan; ^9^ Department of Urology Nagoya University Graduate School of Medicine Nagoya Japan; ^10^ Department of Urology Nara Prefecture General Medical Center Nara Japan; ^11^ Department of Urology, Faculty of Medicine Kyorin University Mitaka Japan; ^12^ Department of Urology, Graduate School of Medicine Hokkaido University Sapporo Japan; ^13^ Department of Urology Kanto Rosai Hospital Kawasaki Japan; ^14^ Department of Pathology, Division of Molecular and Genomic Pathology, Graduate School of Medicine Kobe University Kobe Japan; ^15^ Department of Interstitial Cystitis Medicine, Faculty of Medicine Kyorin University Mitaka Japan

**Keywords:** bladder pain syndrome, hydrodistension, interstitial cystitis, real‐world survey, transurethral surgery

## Abstract

**Objectives:**

To investigate real‐world practices in transurethral surgeries for bladder pain syndrome (BPS) and interstitial cystitis (IC) in Japan, with a focus on procedural characteristics of hydrodistension (HD) and transurethral elimination of Hunner lesions (TUEH).

**Methods:**

An internet‐based questionnaire was sent to all members of the Society of Interstitial Cystitis of Japan in November 2024. The survey inquired about institutional characteristics, the number of procedures performed in the previous 12 months, and detailed surgical techniques. BPS was defined by the absence of Hunner lesions, and IC by their presence. Responses were analyzed to describe procedural patterns and compare practices between HD and TUEH.

**Results:**

Of 205 eligible members, 86 responded (response rate: 42%). Among them, 52 had performed HD for BPS and 67 had performed TUEH for IC within the study period. Most surgeons distended the bladder to a preset pressure of ≤ 80 cm H_2_O for 3–10 min and repeated the procedure at least twice during HD. At TUEH, coagulation was preferred over resection to eliminate Hunner lesions. HD was frequently co‐performed, though the sequence of elimination and HD varied. Compared to HD, TUEH more often relied on abdominal palpation rather than fixed pressure, used shorter distension time, and involved longer catheter placement.

**Conclusions:**

This nationwide survey highlights contemporary surgical practices for BPS and IC in Japan. These findings may help clinicians evaluate and refine their own approaches to transurethral interventions for these complex conditions.

## 1 Introduction

Bladder pain syndrome (BPS) and interstitial cystitis (IC) are intractable conditions characterized by hypersensitive bladder symptoms such as bladder pain, urinary frequency, and urinary urgency [1, 2, 3]. Treatment options include behavioral modification, stress reduction, physiotherapy, pharmacotherapy, intravesical instillation, neuromodulation, and surgical interventions [1, 2, 3]. Among surgical interventions, transurethral surgeries such as hydrodistension (HD) of the bladder and elimination of Hunner lesions are commonly undertaken. In fact, these surgeries have been shown to be effective in reducing bladder pain and other lower urinary tract symptoms in multiple studies [4, 5, 6, 7, 8, 9, 10, 11, 12, 13], and are recommended by clinical guidelines internationally [1, 2, 3]. However, the real‐world procedures of these surgeries have not been necessarily described in detail; for example, the duration of distension during HD, selection of resection versus coagulation mode when eliminating Hunner lesions, whether HD is performed at the elimination of Hunner lesions, and the sequence of biopsy in relation to HD or elimination. To clarify these uncertainties, we conducted a survey on the real‐world surgical procedures.

## 2 Materials and Methods

A survey on surgical procedures was sent to the members of the Society of Interstitial Cystitis in Japan. The questions consisted of institutional information, the number of endoscopic surgeries performed by the individual during the preceding 12 months, and detailed surgical procedures during the period. In this survey, IC and BPS were not connected (such as IC/BPS) but separated, with IC diagnosed by the presence of Hunner lesions on cystoscopy and BPS by the absence of Hunner lesions. Accordingly, endoscopic surgeries were classified as HD for BPS and transurethral elimination of Hunner lesions (TUEH) for IC. HD is defined as bladder distension using hydrostatic pressure under anesthesia, and TUEH as transurethral elimination of Hunner lesions with or without concomitant HD. The questionnaire was sent via the internet to all members of SICJ (*n* = 205) in November 2024 and collected by the end of the year (Supporting Information S1).

Statistical analysis was performed using the chi‐square test for procedures comparable between HD for BPS and TUEH for IC. A *p*‐value less than 0.05 was regarded as significant.

The survey was approved by the institution (approval number 11523‐(5)).

## 3 Results

A total of 86 members responded to the survey (response rate, 42%). Most respondents belonged to hospitals (83.7%) rather than clinics (16.3%). The respondents experienced variable numbers of HD for BPS and TUEH for IC during the period (Table 1). The following analysis was based on the respondents who had at least one case of surgeries during the period. Note that the percentage in the following text or tables represents the ratio of respondents and not the ratio of patients or surgical sessions.

**TABLE 1:** Number of surgeries in the past 12 months and anesthesia types for HD in BPS and TUEH in IC.

	HD for BPS	TUEH for IC	*p*
Number of endoscopic surgeries performed during the recent 12 months (*n*)	86	86	
0	34 (39.5)	19 (22.1)	
1–4	31 (36.0)	34 (39.5)	
5–9	7 (8.1)	12 (14.0)	
10–24	10 (11.6)	18 (20.9)	
25–49	2 (2.3)	2 (2.3)	
50—	2 (2.3)	1 (1.2)	
Anesthesia for surgeries (*n*)	52	67	0.11
General	24 (46.2)	29 (43.3)	
Spinal	20 (38.5)	34 (50.7)	
Epidural	2 (3.8)	3 (4.5)	
Local	6 (11.5)	1 (1.5)	0.042^a^

Abbreviations: BPS, bladder pain syndrome; HD, hydrodistension; IC, interstitial cystitis; TUEH, transurethral elimination of Hunner lesions.

^a^ Intravesical local anesthesia was compared with all other anesthesia methods between TUFH and HD using Fisher's exact test (two‐sided; *p* = 0.042).

### 3.1 Hydrodistension for BPS

A total of 52 respondents performed at least one case of HD for BPS during the period. Most respondents used general anesthesia (46.2%) or spinal anesthesia (38.5%), and a notable portion (11.5%) used local anesthesia (Table 1).

About two‐thirds (63.5%) of the respondents dilated the bladder to a preset hydrostatic pressure. Most preset pressures were 80 cm H_2_O or less (Table 2). The duration of the bladder distension varied, from 0 min (1.9%) to more than 10 min (3.8%), with durations of more than 3 to 5 min and more than 5 to 10 min being most common (32.7% each). Half of the respondents (50.0%) distended the bladder twice, followed by three times (23.1%) and once (19.2%). More than half of the respondents (57.7%) always took bladder mucosa biopsies at the first H, while only 7.7% did so at repeated sessions. About half of the respondents (57.7%) performed HD first and then biopsy, while one‐third (34.6%) did so in the reverse sequence (Table 3).

**TABLE 2:** Techniques/conditions used for hydrodistension.

	HD for BPS (*N* = 52)	TUEH for IC (*N* = 67)	*p*
Distension control at HD			0.031
Distend to prefixed pressure	33 (63.5)	32 (47.8)	
Less than 80 cmH_2_O	8 (11.9)	8 (15.4)	
80 cm H_2_O	23 (34.3)	23 (34.3)	
More than 80 cm H_2_O	1 (1.5)	1 (1.9)	
Distend to full expansion of the lower abdomen on palpation	19 (36.5)	31 (46.3)	
Unknown	0	4 (6.0)	
Duration of bladder distension			0.32
0 min (no holding)	1 (1.9)	6 (9.0)	0.126^a^
1 min or less	6 (11.5)	5 (7.5)	
3 min or less	9 (17.3)	10 (14.9)	
5 min or less	17 (32.7)	19 (28.4)	
10 min or less	17 (32.7)	22 (32.8)	
More than 10 min	2 (3.8)	1 (1.5)	
Unknown	0	4 (6.0)	
Number of distension procedures			0.43
Once	10 (19.2)	15 (22.4)	
Twice	26 (50.0)	29 (43.3)	
Three times	12 (23.1)	15 (22.4)	
Four or five times	1 (1.9)	1 (1.5)	
Six times or more	3 (5.8)	2 (3.0)	
Unknown	0	5 (7.5)	

Abbreviations: BPS, bladder pain syndrome; HD, hydrodistension; IC, interstitial cystitis; TUEH, transurethral elimination of Hunner lesions.

^a^ Comparison of no‐holding or holding for fixed duration.

**TABLE 3:** Techniques/conditions used for bladder biopsy.

	HD for BPS (*N* = 52)	TUEH for IC (*N* = 67)	*p*
Bladder biopsy at the first surgery			0.13
Never	9 (17.3%)	5 (7.5)	
Less than half cases	7 (13.4%)	5 (7.5)	
More than half cases	5 (9.6%)	4 (6.0)	
Always	30 (57.7%)	53 (79.1)	
Unknown	1 (1.9%)	0	
Bladder biopsy at repeated sessions			0.14
Never	33 (63.5)	29 (43.3)	
Less than half cases	11 (21.1)	14 (20.9)	
More than half cases	3 (5.7)	8 (12.0)	
Always	4 (7.7)	12 (17.9)	
Unknown	1 (1.9)	4 (6.0)	
Sequence of biopsy and HD/TUEH			0.032
Biopsy ⇒ HD/TUEH	18 (34.6)	34 (50.7)	
HD/TUEH ⇒ Biopsy ⇒ HD/TUEH	3 (5.8)	8 (11.9)	
HD/TUEH ⇒ Biopsy	30 (57.7)	21 (31.3)	
Unknown	1 (1.9)	4 (6.0)	
Biopsy sites			—
Hunner lesion and other non‐lesional area	—	30 (44.7)	
Hunner lesion alone	—	31 (46.3)	
Biopsy not performed	—	6 (9.0)	
Duration of post‐operative catheter indwelling			0.082
0 day (no indwelling)	3 (5.8)	2 (3.0)	
1 day (overnight)	30 (57.7)	31 (46.3)	
2 days	11 (21.2)	20 (29.9)	
3–4 days	6 (11.5)	10 (14.9)	
5+ days	1 (1.9)	4 (6.0)	
Unknown	1 (1.9)	0	
Bladder perforation or rupture			1.0
Never	38 (78.1)	48 (71.9)	
Once or more	14 (26.9)	19 (28.4)	
Need for additional surgery required	2 (3.8)	1 (1.5)	
Not required	12 (23.1)	18 (26.9)	

Abbreviations: BPS, bladder pain syndrome; HD, hydrodistension; IC, interstitial cystitis; TUEH, transurethral elimination of Hunner lesions.

Transurethral catheters were most often placed for 1 day (57.7%), followed by 2 days (21.1%), 3–4 days (11.5%), and 0 days (no indwelling) (5.8%). About a quarter of the respondents (26.9%) experienced bladder perforation or rupture during the 12‐month period; however, additional surgical procedures such as suprapubic drainage or bladder closure were required in two respondents (Table 3), each with one such case.

### 3.2 Transurethral Elimination of Hunner Lesions for IC

A total of 67 respondents performed TUEH for IC during the period. About half of the respondents used spinal anesthesia (50.7%), followed by general anesthesia (43.3%) (Table 1). Selection of elimination mode (coagulation or resection) showed variability. Coagulation dominance (all or more than half of lesions were coagulated) was reported by 76.2% of the respondents, resection dominance (all or more than half of lesions were resected) by 13.4%, and equal dominance by 9% (Table 4).

**TABLE 4:** Techniques/conditions used for TUEH.

(*N* = 67)	Number of respondents (%)
Selection of fulguration mode at TUEH	
Coagulation all	32 (47.8)
Coagulation more than half	19 (28.4)
Resection/Coagulation nearly equal	6 (9.0)
Resection more than half	7 (10.4)
Resection all	2 (3.0)
Unknown	1 (1.5)
Concomitant hydrodistension at TUE	
All the cases	48 (71.6)
More than half	13 (19.4)
Less than half	4 (6.0)
Never	2 (3.0)
Sequence of HD and elimination at TUEH	
Elimination ⇒ HD	26 (38.8)
HD ⇒ elimination	29 (43.3)
Not consistent	10 (15.0)
Unknown	2 (3.0)

Abbreviations: BPS, bladder pain syndrome; HD, hydrodistension; IC, interstitial cystitis; TUEH, transurethral elimination of Hunner lesions.

Concomitant HD during TUEH was always performed by 71.6% of respondents and in more than half of cases by 19.4%. The sequence of elimination and HD was inconsistent: elimination followed by HD in 38.8%, HD followed by elimination in 43.3%, and case‐by‐case decisions in 15.0% (Table 4). At HD during TUEH about half (47.8%) of the respondents distended the bladder to a preset hydrostatic pressure. Another half (46.3%) dilated the bladder to full expansion of the lower abdomen as assessed by palpation. The preset pressures were mostly 80 cm H_2_O or less (Table 2). The duration of the bladder distension varied from 0 min (9.0%) to more than 10 min (1.5%), with more than 5 to 10 min being the most common (32.8%). Distension was commonly performed twice (43.3%), followed by once (22.4%) or three times (22.4%) (Table 2).

Most respondents (79.1%) always took bladder mucosa biopsies at the first TUEH, while only 17.9% did so at repeated sessions. Regarding biopsy sites, 46.3% took samples from Hunner lesions alone, and 44.7% from both Hunner lesions and non‐lesional areas (9.0%, unknown). About half of the respondents (50.7%) performed biopsy prior to HD/TUEH, while one‐third (31.3%) did so in the reverse sequence (Table 3).

Transurethral catheters were most often placed for 1 day (46.3%), followed by 2 days (29.9%), 3–4 days (14.9%), and 5 days or longer (6.0%). More than a quarter of the respondents (28.4%) experienced bladder perforation or rupture during TUEH in the surveyed period; however, additional surgical procedures such as suprapubic drainage or bladder closure were required in one case by one respondent (Table 3).

### 3.3 Comparison Between Corresponding Procedures of HD and TUEH

The survey showed generally similar figures for corresponding procedures between the two surgeries, although some differences may be clinically meaningful. Compared to HD, TUEH was associated with less frequent use of intravesical local anesthesia (*p* = 0.042; Table 1), more frequent use of abdominal palpation to monitor bladder distension (*p* = 0.031), more frequent biopsy prior to HD/TUEH (*p* = 0.032), and a trend toward longer catheterization (*p* = 0.082; Table 3). Non‐holding at distension was more frequent in TUEH, but the difference was not statistically significant (*p* = 0.126; Table 2).

### 3.4 Impact of Institutional Case Volume on Surgical Techniques and Complications

When institutions were stratified by case volume based on the number of patients with confirmed Hunner lesions, high‐volume centers demonstrated more standardized surgical practices. During hydrodistension, high‐volume centers more frequently individualized pressure settings using intraoperative findings (46.5%), whereas low‐volume centers relied more on fixed‐pressure protocols (33.3%) and showed greater technique variability. Although high‐volume centers more often reported experience with bladder perforation, the proportion of cases requiring additional intervention was not increased (*p* = 0.27), with detailed technique comparisons provided in Supporting Information S2.

## 4 Discussion

The survey has clarified the current real‐world procedures of endoscopic surgeries for BPS and IC in Japan. We divided the transurethral surgeries into HD for BPS and TUEH for IC; HD refers to distension of the bladder, while TUEH denotes elimination of Hunner lesions with or without distension. BPS and IC are usually combined such as IC/BPS, since the symptoms of these two conditions are indistinguishable. However, recent research findings have revealed that IC is an inflammatory disease probably resulting from autoimmunity, while BPS shows little or no inflammatory reactions in the bladder [14, 15, 16]. These observations mandate biological as well as clinical distinction of BPS and IC; thus the survey was separated for these two conditions.

Based on the survey results, the procedures of HD for BPS can be summarized as follows. Mostly under general or spinal anesthesia and occasionally under local anesthesia, the surgeons distend the bladder to preset pressure, which is most often 80 cm H_2_O or less. Some surgeons rather palpate the lower abdomen to monitor distension of the bladder. The duration of distension is variable, with 3 to 10 min most common. They usually dilate the bladder twice but may repeat it several times. Biopsy is usually taken at the first HD but often omitted at repeated sessions. The timing of biopsy is usually after HD but may be prior to HD. A transurethral catheter is most often placed for 1 or 2 days. They may encounter bladder perforation or rupture; however, additional surgical procedures are rarely needed.

The key procedure of TUEH for IC is elimination of Hunner lesions. The surgeons select coagulation mode rather than resection mode to eliminate the lesions. They usually co‐perform HD, although the sequence of HD and TUEH varies greatly. Compared with HD for BPS, the surgeons control distension by palpating the lower abdomen rather than filling the bladder to fixed pressure. They are also likely to distend the bladder for a shorter time and place the catheter longer. These differences may reflect surgeons' cautious approach to concomitant HD during TUEH for IC compared with HD for BPS. Biopsy was significantly more often taken prior to HD/TUEH in TUEH, probably because of more confident identification of biopsy sites at an earlier phase of surgery.

The current survey results would be compatible with the surgical procedures and techniques described in the previous articles. In case of HD for BPS, the maximum hydro pressure applied was 80 cm H_2_O [6, 17, 18, 19] or 100 cm H_2_O [7, 20]. Distension was held for a few minutes [6], 4 min or longer [17, 18, 20] or minimally 3 h [19]. The procedure was done once [6, 7, 19] or repeated [17, 18, 20].

Concomitant HD during TUEH appeared alike. The maximum hydro pressure was 80 cm H_2_O [5, 6, 8, 10, 12, 13] or 100 cm H_2_O [21]. Distension time was a few minutes [5, 6, 8, 21] or 8–10 min [10, 12, 13]. The procedure was done once [5, 6, 7, 13] or repeated [8, 10, 21]. Sequence of HD and elimination was HD first [7, 8, 13, 21] or elimination first [10]. Otherwise, details of TUEH were not necessarily clearly described in the articles. Elimination mode was selected as coagulation alone [5, 8] or resection alone [4, 10]. Timing of biopsy was before TUEH [5, 12]. Under‐report of these procedures may mean inconsistency among surgeons or among operations by the same surgeon.

Some important clinical questions on HD procedures have been examined. Longer distension time showed no benefits for symptom relief [17, 22]. Multiple repetitions of HD were reported to be safe [7, 18], although prolonged distension caused bladder rupture [19] and bladder necrosis requiring enterocystoplasty [23]. Related to TUEH, bladder contracture post‐surgery is a concern [4]. Repeated TUEH progressively lowered anesthetic maximum bladder capacity [11] but not voiding volume [8, 21]. Almost identical outcomes on efficacy and safety were demonstrated between pure coagulation mode and pure resection mode at elimination in a prospective randomized study [9]. Another randomized study proved significantly larger pain score reduction by adding therapeutic HD to TUEH [12]. The effects of sequence of elimination, distension and biopsy on outcomes appear to be unreported. In previous reports, bladder perforation or rupture during HD/TUEH has been reported only rarely; however, in our survey, 27%–28% of surgeons had experienced bladder perforation. As biopsy is often performed before hydrodistension in Japan, a fragile biopsy site may predispose to perforation during subsequent distension, warranting further evaluation of how procedural sequence affects complications and outcomes. Additionally, the frequency of bladder perforation or rupture did not differ substantially between high‐volume centers and other institutions. This finding suggests that the occurrence of these complications is not simply attributable to differences in surgeon proficiency; rather, it may reflect that high‐volume centers tend to manage more severe cases and adopt more proactive and aggressive treatment strategies to achieve accurate diagnosis and disease assessment.

Previous articles also mention tricks for surgeries. For example, the surgeons dilated the bladder minimally to make identification of Hunner lesions easier [11], prevented over‐distending the bladder [4, 9, 10] or took biopsy last [13] to avoid bladder rupture, marked the border of lesions in advance to elimination [5, 10, 12, 21], resected or coagulated bladder mucosa minimally to prevent post‐surgical bladder contracture [1, 4], eliminated the lesions prior to HD to evade bleeding interfering with identification of lesions [5, 10].

The mechanisms of HD and TUEH for relieving symptoms are largely unknown. The speculations include increase in neuro‐proliferative factors [24] or damage to submucosal nerves [24] by HD‐induced transient tissue ischemia. Elimination of Hunner lesions is expected to remove inflammatory infiltrates or activated nerve endings located in the lesions [4, 6, 22].

The procedures would be formed based on the clinical evidence, peers' suggestions, putative mechanisms, and finally the surgeon's preference and discretion. The current survey results would provide reference figures when the surgeons are engaged in the real surgery and optimize their own procedures.

Limitations of the study include the sample size (*N* = 86) and sampling bias of respondents, who were restricted to Japanese surgeons. Recalling bias and inaccuracy of memories are innate limitations of such survey research. Finally, the results simply describe the surgeons' preference, thus have no implication to recommendation level of specific procedures but should only serve as a reference.

In conclusion, the current survey results have clarified the real‐world surgical procedures of HD for BPS and TUEH for IC. They would be useful as a reference when the surgeons optimize their own procedures.

## Author Contributions

**Niimi Aya:** conceptualization; methodology; software; data curation; formal analysis; investigation; visualization; writing – original draft. **Akiyama Yoshiyuki:** project administration, supervision. **Furuta Akira:** data acquisition, supervision. **Matsuo Tomohiro:** data acquisition, supervision. **Kitta Takeya:** data acquisition, supervision. **Otsuka Atsushi:** data acquisition, supervision. **Mitsui Takahiko:** data acquisition, supervision. **Masumori Naoya:** supervision. **Matsukawa Yoshihisa:** data acquisition, supervision. **Torimoto Kazumasa:** data acquisition, supervision. **Kinjo Manami:** data acquisition, review and editing. **Chiba Hiroki:** review and editing, supervision. **Nomiya Akira:** patient recruitment, supervision. **Maeda Daichi:** supervision. **Homma Yukio:** funding acquisition; supervision; writing – original draft, writing – review and editing.

## Funding

This work was supported by the Ministry of Health, Labour and Welfare (JMPH20FC1013).

## Ethics Statement

The study protocol was approved by the institutional review board of the University of Tokyo (approval no. 11523) and it conforms to the provisions of the Declaration of Helsinki.

## Consent

This study did not involve the collection or use of actual patient data. As this was a questionnaire‐based survey of surgeons regarding surgical procedures, no direct informed consent was obtained from patients.

## Conflicts of Interest

Naoya Masumori is the Editor‐in‐Chief of International Journal of Urology, and Takahiko Mitsui, Yoshiyuki Aiyama, and Manami Kinjyo are Editorial Board members of International Journal of Urology and co‐authors of this article. To minimize bias, they were excluded from all editorial decision‐making related to the acceptance of this article for publication. The remaining authors declare no conflicts of interest.

## Supporting information


**Supporting Information: S1.** Contains the complete survey questionnaire used in this study.

**Supporting Information: S2.** Provides a detailed comparison of surgical technique variations between high and low‐volume centers.

## Data Availability

The data that support the findings of this study are available on request from the corresponding author. The data are not publicly available due to privacy or ethical restrictions.
