# Supplementary material for: A Survey on Real‐World Transurethral Surgery Procedures for Bladder Pain Syndrome and Interstitial Cystitis
Source: Low Urin Tract Symptoms. 2026 Mar 11;18(2):e70058. doi: 10.1111/luts.70058 (PMC12979956; doi:10.1111/luts.70058)
Supplement: Supplementary file 1 — Supporting Information: S1. Contains the complete survey questionnaire used in this study. [file LUTS-18-e70058-s002.docx]

**Supplement S1. Survey Questions**

**Part I. Respondent’s profile and Institutional Characteristics**

Q1. Respondent’s name (not included for analysis)

Q2. Respondent’s e-mail address (not included for analysis)

Q3. Affiliation (not included for analysis)

Q4. What type of facility is your institution (as reported in Q3)?

Response options

- Hospital
- Clinic with inpatient beds
- Outpatient clinic without inpatient beds

Q5. What is the staffing status of anesthesiologists at your institution (as reported in Q3)?

Response options

- Full-time anesthesiologist(s) available
- Only part-time anesthesiologist(s) available
- No anesthesiologist (neither full-time nor part-time)

Q6. Please report the number of patients currently followed at your institution who have confirmed Hunner lesions.

Q7. Please report the number of patients currently followed at your institution who have confirmed absence of Hunner lesions.

Q8. Please report the number of patients currently followed at your institution whose Hunner lesion status is unknown.

**Part II. Transurethral Surgery for Interstitial Cystitis**

Q9. During the past year, how many cases of TUEH did you perform as the primary surgeon for interstitial cystitis?

Instruction: If you select “0 cases,” please skip to Q30.

Response options

- 0 cases
- <5 cases
- ≥5 cases
- ≥10 cases
- ≥25 cases
- ≥50 cases

Q10. What was the standard anesthesia method used for TUEH?

Instruction: If multiple methods were used, select the single primary method.

Response options

- General anesthesia
- Spinal anesthesia
- Epidural anesthesia
- Intravesical local anesthesia

Q11. Approximately what proportion of cases used electrocautery as the energy source during TUEH? *(Assume that non-electrocautery devices refer to laser.)*

Response options

- Almost 0%
- <25%
- ≥25%
- ≥50%
- ≥75%
- Almost 100%
- Unable to answer (e.g., not sure)

Q12. When fulgurating Hunner lesions, which technique was primarily used: resection (TUR) or coagulation (TUC)?

*(Please consider the proportion relative to the lesion area treated.)*

Response options

- Almost entirely TUR
- TUR predominates (more than half of the treatment)
- TUR and TUC were used approximately equally
- TUC predominates (more than half of the treatment)
- Almost entirely TUC
- Unable to answer (e.g., not sure)

Q13. When fulgurating Hunner lesions, what depth was generally targeted?

*(Because Hunner lesions lack the epithelial layer, even the most superficial treatment is considered to reach at least the lamina propria.)*

Response options

- Up to the lamina propria
- Up to the superficial muscular layer
- Up to the deep muscular layer
- Unable to answer (e.g., not sure)

Q14. When TUEH was performed, what proportion of cases also included hydrodistension (HD)?

*(For cases in which HD was not performed as a standalone procedure because TUEH “naturally results in HD,” please count these as “HD not performed”.)*

Instruction: If you answer “Almost 0%,” please answer Q15–Q19 as “Unable to answer.”

Response options

- Almost 0%
- <25%
- ≥25%
- ≥50%
- ≥75%
- Almost 100%
- Unable to answer (e.g., not sure)

Q15. When both TUEH and HD were performed, in what proportion of cases was the sequence TUEH → HD? *(All other cases are considered HD → TUEH.)*

Instruction: If you answered “Almost 0%” in Q14, please select “Unable to answer.”

Response options

- Almost 0%
- <25%
- ≥25%
- ≥50%
- ≥75%
- Almost 100%
- Unable to answer (e.g., not sure)

Q16. When HD was performed, how was the maximum distension determined?

Instruction: If you answered “Almost 0%” in Q14, please select “Unable to answer.”

Response options

- All cases were distended to a fixed water pressure
- Water pressure was individually determined for each case, and distension was performed up to that pressure
- An upper pressure limit was set in advance; however, intraoperative endoscopic findings and abdominal palpation were used to decide distension up to full bladder distension
- Distension was determined by infusion volume set per case based on indicators such as maximum voided volume (rather than water pressure)
- Unable to answer (e.g., not sure)

Q17. If HD was performed to a fixed water pressure, what was the water column pressure?

Instructions

- If you answered “Almost 0%” in Q14, select “Unable to answer.”
- If you answered “All cases were distended to a fixed water pressure” in Q16, select the closest value. Otherwise, select “Not applicable.”

Response options

- 50 cmH₂O
- 60 cmH₂O
- 70 cmH₂O
- 80 cmH₂O
- 80 cmH₂O
- Unable to answer (e.g., not sure)
- Not applicable (selected an option other than “All cases were distended to a fixed water pressure”)

Q18. When HD was performed, how long was the distension maintained?

Instruction: If you answered “Almost 0%” in Q14, please select “Unable to answer.”

Response options

- Distension was not maintained (immediate drainage)
- <1 minute
- ≥1 minute
- ≥3 minutes
- ≥5 minutes
- ≥10 minutes
- Unable to answer (e.g., not sure)

Q19. When HD was performed, how many distension cycles were typically performed?

Instruction: If you answered “Almost 0%” in Q14, please select “Unable to answer.”

Response options

- 1 cycle
- 2 cycles
- 3 cycles
- 5 cycles
- ≥6 cycles
- Unable to answer (e.g., not sure)

Q20. During the first TUEH, in what proportion of cases was bladder biopsy performed?

Response options

- Almost 0%
- <25%
- ≥25%
- ≥50%
- ≥75%
- Almost 100%
- Unable to answer (e.g., not sure)

Q21. During the second and subsequent TUEH procedures, in what proportion of cases was bladder biopsy performed?

Response options

- Almost 0%
- <25%
- ≥25%
- ≥50%
- ≥75%
- Almost 100%
- Unable to answer (e.g., not sure)

Q22. If biopsy was performed, in what proportion of cases was it performed before TUEH or HD?

Instruction: If biopsy was not performed, please select “Unable to answer.”

Response options

- Almost 0%
- <25%
- ≥25%
- ≥50%
- ≥75%
- Almost 100%
- Unable to answer (e.g., not sure)

Q23. If biopsy was performed, in what proportion of cases was it performed during TUEH or HD?

Instruction: If biopsy was not performed, please select “Unable to answer.”

Response options

- Almost 0%
- <25%
- ≥25%
- ≥50%
- ≥75%
- Almost 100%
- Unable to answer (e.g., not sure)

Q24. If biopsy was performed, in what proportion of cases was it performed after TUEH or HD?

Instruction: If biopsy was not performed, please select “Unable to answer.”

Response options

- Almost 0%
- <25%
- ≥25%
- ≥50%
- ≥75%
- Almost 100%
- Unable to answer (e.g., not sure)

Q25. If biopsy was performed, in what proportion of cases was the biopsy taken only from Hunner lesions?

*(All other cases are considered biopsies taken from both Hunner lesions and non-lesional bladder mucosa.)*

Instruction: If biopsy was not performed, please select “Unable to answer.”

Response options

- Almost 0%
- <25%
- ≥25%
- ≥50%
- ≥75%
- Almost 100%
- Unable to answer (e.g., not sure)

Q26. After TUEH, how long was a urinary catheter typically left in place?

*(Assuming no bladder rupture or perforation occurred.)*

Response options

- 0 days (removed on the same day / not placed)
- 1 day (removed the next day)
- 2 days
- 3–4 days
- 5–6 days

Q27. In what proportion of cases was the operative record for TUEH documented following the example described in the clinical practice guidelines?

Response options

- Almost 0%
- <25%
- ≥25%
- ≥50%
- ≥75%
- Almost 100%
- Unable to answer (e.g., not sure)

Q28. During TUEH, in what proportion of cases did bladder perforation or bladder rupture occur?

Response options

- 0%
- <2%
- ≥2%
- ≥5%
- ≥10%
- ≥25%

Q29. During TUEH, how many cases of bladder perforation or rupture required additional interventions such as drainage, treatment for peritonitis, or open surgical bladder closure?

Response options

- 0 case
- 1 case
- 2 cases
- 3-4 cases
- 5-9 cases
- ≥10 cases
- Unable to answer (e.g., not sure)

Q30. How many cases of TUEH have you participated in to date, as either the primary surgeon or supervising assistant?

Response options

- <5 cases
- ≥5 cases
- ≥10 cases
- ≥25 cases
- ≥50 cases
- ≥100 cases

Q31. Among your answer in Q30, how many cases did you perform as the primary surgeon for TUEH?

Response options

- <5 cases
- ≥5 cases
- ≥10 cases
- ≥25 cases
- ≥50 cases
- ≥100 cases

Q32. To date, as the primary surgeon, in what proportion of TUEH cases have you experienced bladder perforation or rupture?

Response options

- 0%
- <2%
- ≥2%
- ≥5%
- ≥10%

Q33. To date, as the primary surgeon, how many cases of bladder perforation or rupture required additional interventions such as drainage, treatment for peritonitis, or open surgical bladder closure?

Response options

- 0 case
- 1 case
- 2 cases
- 3-4 cases
- 5-9 cases
- ≥10 cases
- Unable to answer (e.g., not sure)

**Part III. Transurethral Surgery for Bladder Pain Syndrome**

Q34. During the past year, how many cases of HD did you perform as the primary surgeon for bladder pain syndrome?

Instruction: If you select “0 cases,” please skip to Q51.

Response options

- 0 cases
- <5 cases
- ≥5 cases
- ≥10 cases
- ≥25 cases
- ≥50 cases

Q35. What was the standard anesthesia method used for HD?

*(If multiple methods were used, select the single primary method.)*

Response options

- General anesthesia
- Spinal anesthesia
- Epidural anesthesia
- Intravesical local anesthesia

Q36. When HD was performed, how was the maximum distension determined?

Response options

- All cases were distended to a fixed water pressure
- Water pressure was individually determined for each case, and distension was performed up to that pressure
- An upper pressure limit was set in advance; however, intraoperative endoscopic findings and abdominal palpation were used to decide distension up to full bladder distension
- Distension was determined by infusion volume set per case based on indicators such as maximum voided volume (rather than water pressure)

Q37. If HD was performed to a fixed water pressure, what was the water column pressure? *(Select the closest value.)*

Instruction

- If you answered “All cases were distended to a fixed water pressure” in Q36, select the closest value; otherwise, select “Not applicable.”

Response options

- 50 cmH₂O
- 60 cmH₂O
- 70 cmH₂O
- 80 cmH₂O
- 80 cmH₂O
- Unable to answer (e.g., not sure)
- Not applicable (selected an option other than “All cases were distended to a fixed water pressure”)

Q38. How long was the distension maintained?

Response options

- Distension was not maintained (immediate drainage)
- <1 minute
- ≥1 minute
- ≥3 minutes
- ≥5 minutes
- ≥10 minutes

Q39. How many distension cycles were typically performed?

Response options

- 1 cycle
- 2 cycles
- 3 cycles
- 5 cycles
- ≥6 cycles

Q40. During the first HD, in what proportion of cases was bladder biopsy performed?

Response options

- Almost 0%
- <25%
- ≥25%
- ≥50%
- ≥75%
- Almost 100%
- Unable to answer (e.g., not sure)

Q41. During the second and subsequent HD procedures, in what proportion of cases was bladder biopsy performed?

Response options

- Almost 0%
- <25%
- ≥25%
- ≥50%
- ≥75%
- Almost 100%
- Unable to answer (e.g., not sure)

Q42. If biopsy was performed, in what proportion of cases was it performed before HD?

Instruction: If biopsy was not performed, please select “Unable to answer.”

Response options

- Almost 0%
- <25%
- ≥25%
- ≥50%
- ≥75%
- Almost 100%
- Unable to answer (e.g., not sure)

Q43. If biopsy was performed, in what proportion of cases was it performed during HD?

Instruction

- If biopsy was not performed, please select “Unable to answer.”

Response options

- Almost 0%
- <25%
- ≥25%
- ≥50%
- ≥75%
- Almost 100%
- Unable to answer (e.g., not sure)

Q44. If biopsy was performed, in what proportion of cases was it performed after HD?

Instruction: If biopsy was not performed, please select “Unable to answer.”

Response options

- Almost 0%
- <25%
- ≥25%
- ≥50%
- ≥75%
- Almost 100%
- Unable to answer (e.g., not sure)

Q45. If biopsy was performed, in what proportion of cases was hemostasis required for bleeding from the biopsy site?

Instruction: If biopsy was not performed, please select “Unable to answer.”

Response options

- Almost 0%
- <25%
- ≥25%
- ≥50%
- ≥75%
- Almost 100%
- Unable to answer (e.g., not sure)

Q46. Regardless of whether biopsy was performed, in what proportion of cases was hemostasis required for bladder bleeding from sites other than the biopsy site?

Response options

- Almost 0%
- <25%
- ≥25%
- ≥50%
- ≥75%
- Almost 100%
- Unable to answer (e.g., not sure)

Q47. After HD, how long was a urinary catheter typically placed?

Response options

- 0 days (removed on the same day / not placed)
- 1 day (removed the next day)
- 2 days
- 3–4 days
- 5–6 days
- Unable to answer (e.g., not sure)

Q48. In the operative record for HD, in what proportion of cases was the status of post-distension mucosal bleeding documented following the example described in the clinical practice guidelines?

Response options

- Almost 0%
- <25%
- ≥25%
- ≥50%
- ≥75%
- Almost 100%
- Unable to answer (e.g., not sure)

Q49. During HD, in what proportion of cases did bladder perforation or bladder rupture occur?

Response options

- 0%
- <2%
- ≥2%
- ≥5%
- ≥10%
- ≥25%
- Unable to answer (e.g., not sure)

Q50. During HD, how many cases of bladder perforation or rupture required additional interventions such as abdominal drainage, treatment for peritonitis, or open surgical bladder closure?

Response options

- 0 case
- 1 case
- 2 cases
- 3-4 cases
- 5-9 cases
- ≥10 cases
- Unable to answer (e.g., not sure)

Q51. How many cases of HD have you participated in to date, as either the primary surgeon or supervising assistant?

Response options

- <5 cases
- ≥5 cases
- ≥10 cases
- ≥25 cases
- ≥50 cases
- ≥100 cases
- Unable to answer (e.g., not sure)

Q52. Among your answer in Q51, how many cases did you perform as the primary surgeon for HD?

Response options

- <5 cases
- ≥5 cases
- ≥10 cases
- ≥25 cases
- ≥50 cases
- ≥100 cases
- Unable to answer (e.g., not sure)

Q53. To date, as the primary surgeon, in what proportion of HD cases have you experienced bladder perforation or rupture?

Response options

- 0%
- <2%
- ≥2%
- ≥5%
- ≥10%
- ≥25%
- Unable to answer (e.g., not sure)

Q54. To date, as the primary surgeon, how many cases of bladder perforation or rupture required additional interventions such as abdominal drainage, treatment for peritonitis, or open surgical bladder closure?

Response options

- 0 case
- 1 case
- 2 cases
- 3-4 cases
- 5-9 cases
- ≥10 cases
- Unable to answer (e.g., not sure)

**Part IV. Cystectomy and Urinary Diversion**

Q55. How many cases of cystectomy (total or partial) for interstitial cystitis have you performed as the primary surgeon?

Response options

- 0 case
- 1 case
- 2–3 cases
- 4–6 cases
- ≥7 cases
- Unable to answer (e.g., not sure)

Q56. How many cases of urinary diversion without cystectomy for interstitial cystitis have you performed as the primary surgeon?

Response options

- 0 case
- 1 case
- 2–3 cases
- 4–6 cases
- ≥7 cases
- Unable to answer (e.g., not sure)

Q57. How many cases of cystectomy (total or partial) for bladder pain syndrome have you performed as the primary surgeon?

Response options

- 0 case
- 1 case
- 2–3 cases
- 4–6 cases
- ≥7 cases
- Unable to answer (e.g., not sure)

Q58. How many cases of urinary diversion without cystectomy for bladder pain syndrome have you performed as the primary surgeon?

Response options

- 0 case
- 1 case
- 2–3 cases
- 4–6 cases
- ≥7 cases
- Unable to answer (e.g., not sure)
