# Supplementary material for: A Survey on Real‐World Transurethral Surgery Procedures for Bladder Pain Syndrome and Interstitial Cystitis
Source: Low Urin Tract Symptoms. 2026 Mar 11;18(2):e70058. doi: 10.1111/luts.70058 (PMC12979956; doi:10.1111/luts.70058)
Supplement: Supplementary file 2 — Supporting Information: S2. Provides a detailed comparison of surgical technique variations between high and low‐volume centers. [file LUTS-18-e70058-s001.docx]

# Supplement S2 : Detailed comparison of surgical technique variation between high- and low-volume centers

Definition: High-volume centres were defined as institutions following ≥10 patients with confirmed Hunner lesions (Q6). Low-volume centres followed <10 patients.

1. Hydrodistension (HD): pressure setting and distension protocol for IC

| Variable | High-volume centres (n=51) | Low-volume centres (n=35) |
| --- | --- | --- |
| Fixed water pressure for all cases | 20 (46.5%) | 8 (33.3%) |
| Upper pressure limit + intraoperative assessment | 20 (46.5%) | 10 (41.7%) |
| Individually determined pressure | 2 (4.7%) | 2 (8.3%) |
| Volume-based determination | 1 (2.3%) | 0 (0%) |
| Unable to answer | 0 (0%) | 4 (16.7%) |

## 2. TUEH: fulguration technique and depth

| Variable | High-volume centres (n=51) | Low-volume centres (n=35) |
| --- | --- | --- |
| Almost entirely TUR | 2 (4.7%) | 0 (0%) |
| TUR-predominant | 2 (4.7%) | 5 (20.8%) |
| Balanced TUR and TUC | 4 (9.3%) | 2 (8.3%) |
| TUC-predominant | 15 (34.9%) | 4 (16.7%) |
| Almost entirely TUC | 19 (44.2%) | 13 (54.2%) |
| Up to lamina propria | 31 (72.1%) | 16 (66.7%) |
| Up to superficial muscular layer | 10 (23.3%) | 5 (20.8%) |
| Up to deep muscular layer | 1 (2.3%) | 2 (8.3%) |

## 3. Biopsy practice and procedural sequencing

| Variable | High-volume centres (n=51) | Low-volume centres (n=35) |
| --- | --- | --- |
| Biopsy before procedure (Almost 0%) | 13 (30.2%) | 10 (41.7%) |
| Biopsy before procedure (Almost 100%) | 18 (41.9%) | 10 (41.7%) |
| Biopsy during procedure (Almost 0%) | 28 (65.1%) | 15 (62.5%) |
| Biopsy during procedure (Almost 100%) | 2 (4.7%) | 5 (20.8%) |
| Biopsy after procedure (Almost 0%) | 20 (46.5%) | 15 (62.5%) |
| Biopsy after procedure (Almost 100%) | 11 (25.6%) | 6 (25.0%) |
